# Neoadjuvant Immunotherapy in Localized dMMR/MSI-H Colon Cancer: A Systematic Review of Pathological Response and Surgical Outcomes

**DOI:** 10.1245/s10434-026-20430-9

**Published:** 2026-08-17

**Authors:** Rathin Gosavi, Baxter Smith, Hanumant Chouhan, Thang Chien Nguyen, William Teoh, Paul McMurrick, Vignesh Narasimhan

**Affiliations:** 1 Department of Surgery (School of Clinical Sciences at Monash Health), Monash University, Melbourne, VIC Australia; 2 Department of Colorectal Surgery, Monash Health, Melbourne, VIC Australia; 3 Department of Colorectal Surgery, Cabrini Health, Melbourne, VIC Australia; 4 Department of Colorectal Surgery, Alfred Health, Melbourne, VIC Australia

**Keywords:** Colon cancer, dMMR, MSI-H, Neoadjuvant immunotherapy, Immune checkpoint inhibition, Pathological complete response

## Abstract

**Background:**

Neoadjuvant immune checkpoint inhibition is increasingly being investigated in localized mismatch repair-deficient or microsatellite instability-high (dMMR/MSI-H) colon cancer, but evidence remains immature and often combined with broader cohorts.

**Methods:**

We reviewed prospective interventional studies of neoadjuvant immune checkpoint inhibition in adults with localized, non-metastatic dMMR/MSI-H colon cancer. The Ovid MEDLINE, Embase, Web of Science, Cochrane Library, and ClinicalTrials.gov databases and reference lists were searched to 12 June 2026. Studies were eligible when colon-specific pathological response outcomes were reported or extractable. Primary outcomes were pathological complete response (pCR) and major pathological response (MPR). Secondary outcomes included surgical feasibility, R0 resection, delay, morbidity, adverse events, recurrence, and survival.

**Results:**

Six studies were included: NICHE, NICHE-2, NICHE-3, RESET-C, IMHOTEP, and IBI310/sintilimab. pCR ranged from 44 to 78.4% and MPR from 57 to 95%, varying by regimen, denominator, and definition. NICHE-based dual-checkpoint studies reported pCR rates of 60–68% and MPR rates of 92–95%. Pembrolizumab-based studies reported pCR rates of 44–62.7%. The only randomized comparative study favored CTLA-4 plus programmed cell death protein-1 blockade over programmed cell death protein-1 blockade alone. Most patients proceeded to colectomy, with high R0 resection rates where reported. Severe treatment-related or immune-related adverse events occurred, including rare grade 5 events. Follow-up ranged from 8.1 to 26 months; recurrence was uncommon and survival outcomes immature.

**Conclusions:**

Neoadjuvant immune checkpoint inhibition demonstrates substantial pathological activity and apparent surgical feasibility in selected localized dMMR/MSI-H colon cancer. Longer follow-up and comparative trials are needed to define regimen, timing, patient selection, and durable oncological benefit.

**Supplementary Information:**

The online version contains supplementary material available at https://doi.org/10.1245/s10434-026-20430-9.

Immune checkpoint inhibitors have changed the treatment landscape for mismatch repair-deficient (dMMR) and microsatellite instability-high (MSI-H) colorectal cancer. This molecular subgroup is characterized by high tumor mutational burden, increased neoantigen load, and an immune-inflamed tumor microenvironment, providing a strong biological rationale for programmed cell death protein-1 blockade, either alone or in combination with other checkpoint inhibitors.^1,2^ Although this benefit was first established in metastatic disease,^3,4^ there is increasing interest in moving immunotherapy into the neoadjuvant setting for patients with localized disease.

For localized colon cancer, surgery remains the cornerstone of curative treatment. However, patients with dMMR/MSI-H tumors derive less consistent benefit from conventional fluoropyrimidine-based chemotherapy, and pathological response to neoadjuvant chemotherapy appears limited in this subgroup.^5,6^ Neoadjuvant immunotherapy offers a different strategy by exploiting tumor immunogenicity while allowing pathological response to be assessed directly in the resected specimen.

The clinical priorities in colon cancer differ from those in rectal cancer. In rectal cancer, much of the interest in neoadjuvant immunotherapy relates to clinical complete response, watch-and-wait management, and avoidance of pelvic surgery.^7,8^ In colon cancer, organ preservation is less established. The more immediate questions are whether neoadjuvant immunotherapy can induce pathological complete response (pCR) or major pathological response (MPR), whether colectomy remains safe and timely, and whether deep pathological response may predict durable disease control.

Several prospective studies have now evaluated neoadjuvant immune checkpoint inhibition in localized dMMR/MSI-H colon cancer, including dual checkpoint blockade, single-agent programmed cell death protein-1 (PD-1) blockade and perioperative strategies.^9–14^ These studies vary in design, treatment duration, pathological response definitions, surgical reporting, and follow-up. This review builds on prior colon-specific and broader colorectal cancer syntheses^15,16^ by focusing on updated prospective colon cancer-specific pathological, surgical, and early oncological outcomes.

This systematic review aimed to evaluate currently available prospective evidence for neoadjuvant immune checkpoint inhibition in localized dMMR/MSI-H colon cancer. The primary outcomes were pCR and MPR. Secondary outcomes included surgical feasibility, R0 resection, perioperative morbidity, treatment-related toxicity, and early oncological outcomes.

## Materials and Methods

### Search Strategy

This systematic review was performed in accordance with the Preferred Reporting Items for Systematic Reviews and Meta-Analyses (PRISMA) guidelines. A comprehensive literature search was conducted using Ovid MEDLINE, Embase, Web of Science, and the Cochrane Library databases from inception to 12 June 2026. ClinicalTrials.gov and reference lists of relevant articles were also searched. Search terms included combinations and variations of “colon cancer,” “colorectal cancer,” “dMMR,” “mismatch repair deficient,” “MSI-H,” “microsatellite instability,” “neoadjuvant,” “preoperative,” “immunotherapy,” “immune checkpoint inhibitor,” “PD-1,” “PD-L1,” “CTLA-4,” “nivolumab,” “ipilimumab,” “pembrolizumab,” “relatlimab,” “sintilimab,” “atezolizumab,” and “toripalimab.” The full search strategy is provided in Supplementary Table 1.

### Study Selection

Eligible studies were prospective interventional studies evaluating immune checkpoint inhibitors as neoadjuvant treatment for adults with localized, non-metastatic dMMR/MSI-H colon adenocarcinoma. Studies were included if immune checkpoint blockade was administered before planned curative-intent colectomy and if pathological response outcomes were reported or extractable. Surgical outcomes were extracted when reported. Single-arm, randomized non-comparative and comparative prospective studies were eligible.

Studies enrolling mixed colorectal cancer populations were included only when colon cancer-specific pathological response outcomes were separately reported or extractable; surgical outcomes were extracted where separately available. Studies reporting outcomes only for combined colorectal cohorts were excluded from the primary synthesis and summarized narratively where relevant. Retrospective studies, case reports, reviews, editorials, metastatic-only studies, rectal cancer-only studies, pan-tumor studies without colon-specific outcomes, and studies without a neoadjuvant immunotherapy component were excluded. Conference abstracts were screened but were not included in the primary synthesis unless sufficient outcome data were available.

Two reviewers (RG and BS) independently screened titles, abstracts, and full-text articles. Disagreements were resolved by consensus or by consultation with the senior author. Where multiple reports described the same trial, the most recent and complete report was used as the primary source, with earlier reports and supplementary appendices used to clarify missing data.

### Quality Assessment

The methodological quality of included studies was assessed using the Methodological Index for Non-Randomized Studies. Non-comparative studies were scored out of 16 and comparative studies out of 24 (Supplementary Table 2). Quality assessment was performed independently by two reviewers, with disagreements resolved by consensus.

### Outcomes

The primary outcomes were pCR and MPR. pCR was defined according to each study’s reported definition, most commonly absence of viable tumor in the primary tumor and sampled lymph nodes. MPR was defined as residual viable tumor of ≤10% or according to the definition used by each study.

Secondary outcomes were overall pathological response, R0 resection, proceeded-to-surgery rate, treatment-related surgical delay, postoperative morbidity, grade 3 or higher treatment-related or immune-related adverse events, recurrence, disease-free survival, event-free survival, and overall survival. Treatment-related surgical delay was extracted according to each study’s reported definition. Where reported, this reflected postponement of planned surgery attributed to treatment-related toxicity. Recurrence, disease-free survival, event-free survival, and overall survival were extracted using each study’s reported definitions, assessment time points, and follow-up duration.

### Data Extraction and Synthesis

Data were extracted independently by two reviewers using a predefined data extraction form. Extracted variables included study design, trial registration, sample size, patient demographics, molecular status, neoadjuvant regimen, treatment sequence, pathological response, surgical outcomes, adverse events, and follow-up.

Given the clinical and methodological heterogeneity across studies, formal meta-analysis was not performed. Results were synthesized narratively and presented in tables. Mixed colorectal studies were included only when colon-specific pathological and surgical outcomes were separately extractable.

## Results

### Search Results

**Figure 1:**
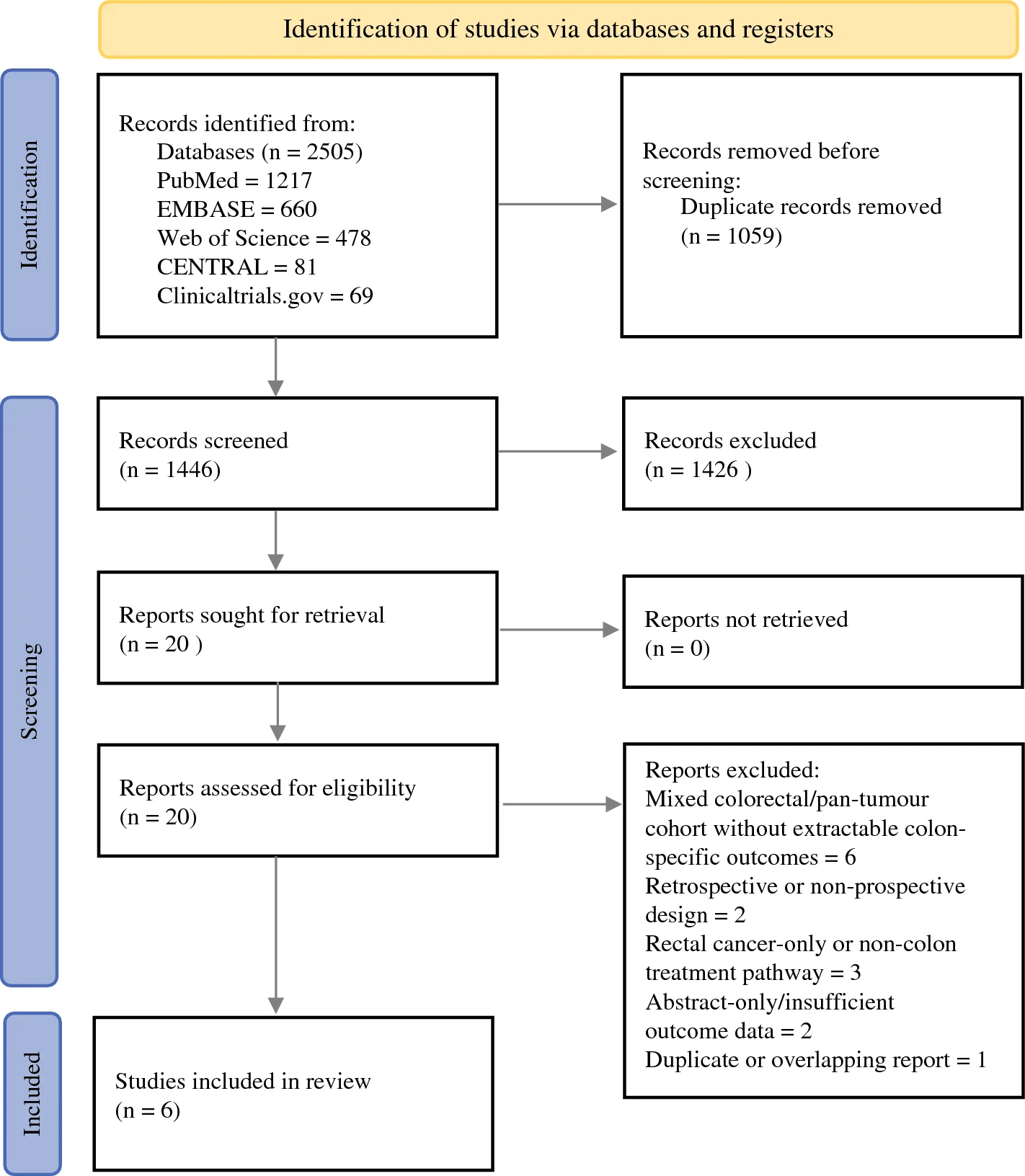
Preferred reporting items for systematic reviews and meta-analyses (PRISMA) flow diagram of study selection

The initial database and register search identified 2505 records. After removal of 1059 duplicates, 1446 records underwent title and abstract screening. A total of 20 reports were sought for retrieval and assessed for eligibility, of which 14 were excluded. Six studies met the inclusion criteria for qualitative synthesis: NICHE, NICHE-2, NICHE-3, RESET-C, IMHOTEP, and the IBI310/sintilimab randomized phase Ib trial.^9–14^ The PRISMA flow diagram is shown in Fig. 1.

### Study Characteristics

All included studies were prospective early-phase studies evaluating neoadjuvant immune checkpoint inhibition in localized dMMR/MSI-H colon cancer cohorts. Five were phase II studies and one was a randomized phase Ib trial. Publication years ranged from 2020 to 2026, and studies were conducted in the Netherlands, Denmark, France, and China.

Neoadjuvant regimens included dual checkpoint inhibition with ipilimumab plus nivolumab, nivolumab plus relatlimab, IBI310 plus sintilimab, single-agent pembrolizumab, and sintilimab monotherapy. Treatment duration ranged from short-course preoperative regimens to perioperative strategies incorporating postoperative immunotherapy. The IBI310/sintilimab study was the only randomized comparative trial, evaluating dual cytotoxic T-lymphocyte-associated protein (CTLA)-4 plus PD-1 blockade against PD-1 blockade alone in locally advanced MSI-H/dMMR colon cancer. Study characteristics are summarized in Table 1.

**Table 1:** Characteristics of included prospective studies evaluating neoadjuvant immune checkpoint inhibition in localised dMMR/MSI-H colon cancer

Study, year, country	Study design (trial number)	Sample size included	Male/female	Age, years^a^	Molecular status	Population	ICI	Control	Treatment modality and sequence
Chalabi et al.,^9^ NICHE, 2020, Netherlands	Prospective, exploratory phase II study (NCT03026140)	21 dMMR colon tumors; 20 efficacy evaluable	9/12	58.4 (22–82)	dMMR subgroup	Stage I–III colon cancer	IPI + NIV	No	IPI D1 plus NIV D1 and D15, followed by surgery within 6 wk
Chalabi et al.,^10^ NICHE-2, 2024, Netherlands	Prospective, multicenter, single-arm phase II study (NCT03026140)	115 enrolled; 111 efficacy evaluable	48/67	60 (20–82)	dMMR	LA non-metastatic colon cancer	IPI + NIV	No	IPI D1 plus NIV D1 and D15, followed by planned surgery within 6 wk
de Gooyer et al.,^11^ NICHE-3, 2024, Netherlands	Prospective, single-arm phase II study (NCT03026140)	59	27/32	65 (21–85)	dMMR	LA non-metastatic colon cancer	NIV + REL	No	Two 4-wkly cycles of NIV + REL, followed by surgery within 8 wk of enrolment
Gogenur et al.,^12^ RESET-C, 2026, Denmark	Prospective, national multicenter phase II study (NCT05662527)	85 enrolled; 84 surgical efficacy evaluable	24/61	74 (IQR 68–79)	dMMR	Localized stage I–III colon cancer	PEM	No	Single-cycle PEM, followed by endoscopy and surgery 3–5 wk later
de la Fouchardière et al.,^13^ IMHOTEP, 2026, France	Prospective, multicenter, non-randomized basket phase II study (NCT04795661)	63 colon cancers in efficacy population	CRC efficacy cohort: 32/40	CRC efficacy cohort: 66 (21–89)	dMMR/MSI	Localized resectable dMMR/MSI CRC; colon-specific data extracted	PEM	No	One or two cycles of PEM before surgery, followed by postoperative PEM to complete 1 year where administered
Wang et al.,^14^ IBI310 + sintilimab, 2025, China	Prospective, multicenter, randomized phase Ib study (NCT05890742)	101 randomized; 96 mITT surgical population	56/45	56 (23–77)	MSI-H/dMMR	LA colon cancer	IBI310 + SIN vs. SIN	SIN monotherapy	Two cycles of neoadjuvant treatment followed by surgery

^a^Age is presented as median (range) unless otherwise indicated

CRC, colorectal cancer; D, day; dMMR, mismatch repair deficient; ICI, immune checkpoint inhibitor; IPI, ipilimumab; IQR, interquartile range; LA, locally advanced; mITT, modified intent to treat; MSI-H, microsatellite instability-high; NIV, nivolumab; NR, not reported; PEM, pembrolizumab; REL, relatlimab, SIN, sintilimab; wk, week(s)

### Pathological Response

Primary and secondary outcomes are summarized in Table 2. pCR and MPR were the primary outcomes of this review. Definitions varied between studies. Most studies defined pCR as absence of viable tumor in the primary tumor and lymph nodes, whereas RESET-C^12^ defined pCR according to Mandard tumor regression grade 1 and did not include lymph node status in the primary pCR definition.

**Table 2:** Primary and secondary outcomes of included prospective studies

Study	Pathological response denominator	pCR	MPR	Overall pathological response	Surgery performed	R0 resection	Surgical delay	Postoperative morbidity	Grade ≥3 TRAE/irAE	Median follow-up	Recurrence/survival
Chalabi et al.,^9^ NICHE	20 dMMR efficacy-evaluable tumors	12/20 (60%)	19/20 (95%)	20/20 (100%)	All underwent radical resection within 6 weeks	Radical resection in all	None reported	Surgery-related grade 3 AEs in 8/40 overall cohort; anastomotic leak 4/40 overall cohort	Grade 3–4 treatment-related toxicity 5/40 overall cohort	8.1 mo in dMMR subgroup	All dMMR pts alive and disease-free at data cut-off
Chalabi et al., ^10^ NICHE-2	111 efficacy-evaluable pts	75/111 (68%)	105/111 (95%)	109/111 (98%)	115/115 (100%)	115/115 (100%)	>2-wk delay in 2/115	Surgery-related AEs any grade 22/115; grade 3 surgery-related AEs 12/115; anastomotic leak 4/115	Grade 3–4 irAEs 5/115 (4%)	26 mo	No recurrence reported
de Gooyer et al., ^11^ NICHE-3	59 pts	40/59 (68%)	54/59 (92%)	57/59 (97%)	59/59 (100%)	59/59 (100%)	Treatment-related delay in 3/59	Grade 3–4 postoperative AEs 4/59, including ileus, SSI, intra-abdominal abscess, anastomotic leak, postoperative hemorrhage	Grade 3–4 irAEs 6/59 (10%)	8 mo	One recurrence reported
Gogenur et al., ^12^ RESET-C	84 surgical efficacy-evaluable pts	37/84 (44%)	48/84 (57%)	NR	84/85 (99%)	NR	NR	Surgical complications 31/84; Clavien–Dindo ≥3b 8/84; median LOS 4 days; readmission 4/84; two deaths within 30 days of surgery	Grade 3 AEs 9/85 (11%); three treatment-related grade 3 events	18.4 mo	One recurrence; OS 98%, DFS 96%
de la Fouchardière et al., ^13^ IMHOTEP	63 colon efficacy pts for ypT0N0 pCR; 59 colon pts for central RVT assessment	ypT0N0 pCR 35/63 (55.6%); central RVT-based pCR 37/59 (62.7%)	43/59 (72.9%); near-complete response 6/59 (10.2%)	NR separately	CRC efficacy cohort: 72/72 underwent surgery	72/72 (100%)	NR	Postoperative Clavien–Dindo complications in 13/78 operated pts	Grade ≥3 TRAEs 14/89 (15.7%); one grade 5 PEM-related myasthenia	24.5 mo	Three recurrences in operated CRC cohort; 24-mo OS 98%
Wang et al., ^14^ IBI310 + SIN	mITT: 51 combination; 45 monotherapy	IBI310 + sintilimab: 40/51 (78.4%); sintilimab: 21/45 (46.7%)	IBI310 + SIN: 48/51 (94.1%); SIN: 33/45 (73.3%)	NR	51/52 combination; 45/49 monotherapy	96/96 (100%)	2 in combination arm; 1 in monotherapy arm	NR in main outcomes table	Grade ≥3 TRAE: 2/52 combination; 5/49 monotherapy; one grade 5 myocarditis in monotherapy arm	21.4 mo	No recurrence reported

AE, adverse event; CRC, colorectal cancer; CSS, cancer-specific survival; DFS, disease-free survival; dMMR, mismatch repair deficient; irAE, immune-related AE; LOS, length of stay; mITT, modified intent to treat; mo, month(s); MPR, major pathological response; NR, not reported; OS, overall survival; pCR, pathological complete response; PEM, pembrolizumab; pts, patients; RVT, residual viable tumor; SIN, sintilimab; SSI, surgical site infection; TRAE, treatment-related adverse event

For IMHOTEP, central MPR was defined as ≤10% residual viable tumor and includes pCR; near-complete response refers to 1–10% residual viable tumor

Across included studies, pCR rates ranged from 44 to 78.4%, depending on regimen, denominator, and definition. MPR rates ranged from 57 to 95%. In NICHE-based dual-checkpoint inhibition studies, pCR ranged from 60 to 68%, and MPR ranged from 92 to 95%. Among pembrolizumab-based studies, pCR ranged from 44 to 62.7%, depending on the definition and denominator used. In the randomized IBI310/sintilimab trial, pCR was 78.4% with IBI310 plus sintilimab and 46.7% with sintilimab alone in the modified intention-to-treat population; corresponding MPR rates were 94.1 and 73.3%.

### Surgical Outcomes

All included studies planned curative-intent surgery after neoadjuvant treatment, although one patient in RESET-C declined surgery. Planned timing to surgery varied across protocols, from 3–5 weeks after single-cycle pembrolizumab in RESET-C to within 8 weeks of enrolment in NICHE-3; NICHE and NICHE-2 scheduled surgery within 6 weeks, IMHOTEP used one or two cycles before surgery, and IBI310/sintilimab used two neoadjuvant cycles before surgery. Surgery was performed in all patients in NICHE, NICHE-2, and NICHE-3, in 84 of 85 patients in RESET-C, in 72 patients in the IMHOTEP colorectal efficacy cohort, and in 51 of 52 patients in the IBI310/sintilimab combination arm and 45 of 49 patients in the sintilimab monotherapy arm. R0 resection was reported in all patients in NICHE-2, NICHE-3, IBI310/sintilimab, and the IMHOTEP colorectal efficacy cohort. Surgical delay was uncommon, occurring in two patients in NICHE-2, three patients in NICHE-3, and three patients in the IBI310/sintilimab trial. Postoperative morbidity reporting was heterogeneous. Reported complications included anastomotic leak, ileus, surgical-site or wound infection, intra-abdominal abscess or infection, and postoperative hemorrhage. Grade 3 surgery-related adverse events were reported in 12 of 115 patients in NICHE-2 and 4 of 59 patients in NICHE-3. RESET-C reported surgical complications in 31 of 84 patients, Clavien–Dindo grade ≥3b complications in 8 of 84, a median length of stay of 4 days, and readmission in 4 of 84. IMHOTEP reported postoperative Clavien–Dindo complications in 13 of 78 operated patients. Postoperative morbidity was variably reported and is summarized in Table 2 and Supplementary Table 3.

### Adverse Events

Treatment-related and immune-related adverse events were reported across all included studies, although definitions and reporting windows differed. Grade 3 or higher treatment-related or immune-related adverse events occurred in all studies, ranging from 4% in NICHE-2 to 15.7% in IMHOTEP. NICHE-3 reported grade 3–4 immune-related adverse events in 6 of 59 patients, RESET-C reported grade 3 adverse events in 9 of 85 patients, and the IBI310/sintilimab trial reported grade 3 or higher treatment-related adverse events in 2 of 52 patients in the combination arm and 5 of 49 in the monotherapy arm. Reported immune-related toxicity profiles varied by regimen, with thyroid or endocrine dysfunction among commonly reported events in checkpoint inhibitor studies. Grade 5 events were reported in two studies: pembrolizumab-related myasthenia in IMHOTEP and immune-mediated myocarditis in the sintilimab monotherapy arm of the IBI310/sintilimab trial. Detailed adverse-event and perioperative safety outcomes are presented in Supplementary Table 3.

### Long-Term Outcomes

Follow-up duration ranged from 8.1 to 26 months. No disease recurrence was reported in the dMMR subgroup of NICHE or in NICHE-2 at reported follow-up. One recurrence was reported in NICHE-3 and one in RESET-C; RESET-C reported overall survival of 98% and disease-free survival of 96% at median follow-up of 18.4 months. IMHOTEP reported three recurrences in the operated colorectal cancer cohort and 24-month overall survival of 98%. No disease recurrence was reported in either arm of the IBI310/sintilimab trial at median follow-up of 21.4 months. No included study reported mature comparative overall survival data.

## Discussion

This systematic review identified six prospective studies evaluating neoadjuvant immune checkpoint inhibition in localized dMMR/MSI-H colon cancer cohorts. The principal finding was a consistent and substantial pathological response signal, with pCR rates ranging from 44 to 78.4% and MPR rates ranging from 57% to 95%. Most patients proceeded to curative-intent surgery, and R0 resection rates were high where reported. These findings support neoadjuvant immunotherapy as a highly active strategy in this molecular subgroup, while also highlighting unresolved questions regarding regimen selection, response definitions, surgical reporting, and long-term outcomes.^9–14^

This review differs from broader colorectal cancer syntheses^16^ by focusing specifically on colon cancer. This distinction is clinically important. In rectal cancer, neoadjuvant immunotherapy is often discussed in relation to clinical complete response, watch-and-wait management, and avoidance of pelvic surgery.^17^ In colon cancer, the immediate question is whether neoadjuvant immunotherapy can induce significant pathological response, preserve the safety and timing of colectomy, and improve recurrence outcomes. Combining colon and rectal studies risks conflating different endpoints, treatment intentions, and surgical implications.

Pathological response is therefore the central endpoint in localized dMMR/MSI-H colon cancer. pCR and MPR provide objective measures of treatment effect within the resected specimen and may be useful early markers of systemic disease control.^10^ This is particularly relevant because dMMR/MSI-H colon cancers derive less consistent benefit from conventional fluoropyrimidine-based chemotherapy, and pathological response to neoadjuvant chemotherapy appears limited in this subgroup.^5,6,18^ However, pCR and MPR are not yet validated substitutes for long-term disease-free or overall survival after neoadjuvant immunotherapy.

Pathological response assessment also varied across studies. Most included studies used residual viable tumor thresholds, with MPR generally defined as ≤10% residual viable tumor, whereas RESET-C used Mandard tumor regression grade 1 for pCR and did not include lymph node status in its primary pCR definition. None of the included colon cancer studies explicitly applied immune-related pathological response criteria. This is relevant because conventional regression grading may not fully capture immune-mediated response patterns, including immune activation, tumor cell death, and tissue repair within the regression bed. The immune-related pathological response criteria proposed by Cottrell et al.^19^ provide one framework for quantifying these features, although they were developed in non-small-cell lung cancer and require validation in dMMR/MSI-H colon cancer. Future studies should report residual viable tumor, nodal response, regression-bed features, and the pathological criteria used.

The optimal immunotherapy regimen remains uncertain. Across available prospective studies, dual checkpoint blockade has produced high MPR rates, with MPR rates of 92–95% in NICHE-based dual-agent studies.^10,11^ However, single-agent PD-1 blockade has also produced substantial activity, with pCR rates ranging from 44% to 62.7% in pembrolizumab-based studies.^12,13^ The clearest comparative signal comes from the IBI310/sintilimab trial,^14^ in which CTLA-4 plus PD-1 blockade was associated with higher pCR and MPR than PD-1 blockade alone. Importantly, this trial did not show higher grade 3 or higher treatment-related or immune-related adverse events with combination therapy, although any-grade treatment-related and immune-related adverse events were more frequent with the doublet.^14^ Cross-trial comparisons remain limited by differences in patient selection, baseline stage, treatment duration, timing to surgery, adverse-event reporting windows, and pathological assessment. Future studies will need to define whether selected patients require dual checkpoint blockade, repeated dosing, or whether shorter single-agent regimens are sufficient.

Surgical feasibility is a key consideration because colectomy remains the standard of care for localized colon cancer. Across included studies, most patients proceeded to surgery, and R0 resection was achieved in nearly all patients where reported. Treatment-related surgical delays were uncommon. However, operative and perioperative outcomes were inconsistently reported, including operative approach, conversion, anastomotic leak, stoma formation, readmission, reoperation, and length of stay. This remains a major limitation because pathological response alone does not determine whether a neoadjuvant strategy is safe, practical, or scalable.

Treatment-related surgical delay should also be interpreted carefully. Delay due to immune-related toxicity, clinical deterioration, or loss of a planned operative window is clearly undesirable. However, a longer planned interval to surgery in clinically stable or responding patients may not necessarily be harmful, because immune-mediated tumor regression may continue after cessation of treatment. Rousseau et al.^20^ recently highlighted that longer duration of immunotherapy in mismatch repair-deficient cancers may be associated with a higher incidence of complete response. Future studies should therefore distinguish unplanned toxicity-related delay from protocol-defined or response-adapted extension of the neoadjuvant interval.

Treatment-related toxicity also requires careful consideration. Most adverse events were manageable, but grade 3 or higher immune-related events occurred across several studies, grade 5 immune-related events were reported in two studies, including pembrolizumab-related myasthenia in IMHOTEP^13^ and immune-mediated myocarditis in the sintilimab monotherapy arm of the IBI310/sintilimab trial.^14^ This is particularly relevant in localized disease, where a proportion of patients may already be cured with surgery alone. Future trials should separate neoadjuvant toxicity from postoperative or extended perioperative toxicity and should report whether adverse events delay or compromise surgery.

Long-term oncological outcomes remain immature. Early recurrence rates were low, although follow-up remains short across most included studies. The longest follow-up within the primary synthesis was reported in NICHE-2,^10^ with no recurrence at a median follow-up of 26 months. The IBI310/sintilimab trial^14^ reported no recurrence in either arm at a median follow-up of 21.4 months. However, most studies have shorter follow-up, few recurrence events and limited stratification by pathological response category. Whether patients with pCR or MPR have sufficiently low recurrence risk to justify adjuvant treatment de-escalation remains unknown.

This review has several limitations. Most included studies were phase II trials without surgery-alone control groups. Treatment regimens, duration, and pathological response definitions varied. Surgical morbidity and operative details were inconsistently reported. Finally, follow-up remains limited for several studies, preventing firm conclusions regarding disease-free or overall survival.

A further unresolved question is how neoadjuvant immunotherapy should be integrated into the broader treatment pathway. Current studies demonstrate reproducible pathological downstaging, but whether immunotherapy is best delivered before surgery, after surgery, or as part of a perioperative strategy remains unknown. The phase III ATOMIC trial supports the broader principle that immunotherapy can improve disease-free outcomes in resected stage III dMMR colon cancer, although it evaluated adjuvant mFOLFOX6 with or without atezolizumab rather than neoadjuvant immunotherapy alone, and no statistically significant overall survival advantage was observed at first analysis.^21^ Patient selection is therefore likely to be important: locally advanced, bulky, or borderline resectable tumors may benefit most from downstaging that simplifies surgery or avoids more extensive resection, whereas the risk–benefit balance may differ for lower-risk tumors that are already readily resectable.

The field is now moving from proof-of-concept studies toward randomized phase III evaluation. AZUR-2 is evaluating perioperative dostarlimab monotherapy versus standard of care in patients with untreated T4N0 or stage III resectable dMMR/MSI-H colon cancer.^22^ Such trials should define response rates, optimal timing, regimen duration, surgical impact, and durable recurrence outcomes.

## Conclusion

Neoadjuvant immune checkpoint inhibition demonstrates substantial pathological activity in localized dMMR/MSI-H colon cancer, with high pCR and MPR rates across prospective studies. Curative-intent colectomy appears feasible, with high R0 resection rates where reported. However, the evidence remains limited by early-phase designs, heterogeneous regimens, variable response definitions, and immature long-term outcomes. Further prospective data are required to define treatment timing, patient selection, surgical impact, and whether pathological response translates into durable oncological benefit.

## Supplementary Information

Below is the link to the electronic supplementary material.

Supplementary file1 (DOCX 22 KB)

## References

[1] Llosa NJ, Cruise M, Tam A, Wicks EC, Hechenbleikner EM, Taube JM, et al. The vigorous immune microenvironment of microsatellite instable colon cancer is balanced by multiple counter-inhibitory checkpoints. *Cancer Discov*. 2015;5(1):43–51. 10.1158/2159-8290.CD-14-0863. PMC4293246 25358689

[2] Le DT, Durham JN, Smith KN, Wang H, Bartlett BR, Aulakh LK, et al. Mismatch repair deficiency predicts response of solid tumors to PD-1 blockade. *Science*. 2017;357(6349):409–13. 10.1126/science.aan6733. PMC5576142 28596308

[3] Andre T, Shiu KK, Kim TW, Jensen BV, Jensen LH, Punt C, et al. Pembrolizumab in microsatellite-instability-high advanced colorectal cancer. *N Engl J Med*. 2020;383(23):2207–18. 10.1056/NEJMoa2017699. 33264544

[4] Andre T, Elez E, Lenz HJ, Jensen LH, Touchefeu Y, Van Cutsem E, et al. Nivolumab plus ipilimumab versus nivolumab in microsatellite instability-high metastatic colorectal cancer (CheckMate 8HW): a randomised, open-label, phase 3 trial. *Lancet*. 2025;405(10476):383–95. 10.1016/S0140-6736(24)02848-4. 39874977

[5] Ribic CM, Sargent DJ, Moore MJ, Thibodeau SN, French AJ, Goldberg RM, et al. Tumor microsatellite-instability status as a predictor of benefit from fluorouracil-based adjuvant chemotherapy for colon cancer. *N Engl J Med*. 2003;349(3):247–57. 10.1056/NEJMoa022289. PMC3584639 12867608

[6] Sargent DJ, Marsoni S, Monges G, Thibodeau SN, Labianca R, Hamilton SR, et al. Defective mismatch repair as a predictive marker for lack of efficacy of fluorouracil-based adjuvant therapy in colon cancer. *J Clin Oncol*. 2010;28(20):3219–26. 10.1200/JCO.2009.27.1825. PMC2903323 20498393

[7] Cercek A, Lumish M, Sinopoli J, Weiss J, Shia J, Lamendola-Essel M, et al. PD-1 Blockade in mismatch repair-deficient, locally advanced rectal cancer. *N Engl J Med*. 2022;386(25):2363–76. 10.1056/NEJMoa2201445. PMC9492301 35660797

[8] Cercek A, Foote MB, Rousseau B, Smith JJ, Shia J, Sinopoli J, et al. Nonoperative management of mismatch repair-deficient tumors. *N Engl J Med*. 2025;392(23):2297–308. 10.1056/NEJMoa2404512. PMC12661660 40293177

[9] Chalabi M, Fanchi LF, Dijkstra KK, Van den Berg JG, Aalbers AG, Sikorska K, et al. Neoadjuvant immunotherapy leads to pathological responses in MMR-proficient and MMR-deficient early-stage colon cancers. *Nat Med*. 2020;26(4):566–76. 10.1038/s41591-020-0805-8. 32251400

[10] Chalabi M, Verschoor YL, Tan PB, Balduzzi S, Van Lent AU, Grootscholten C, et al. Neoadjuvant immunotherapy in locally advanced mismatch repair-deficient colon cancer. *N Engl J Med*. 2024;390(21):1949–58. 10.1056/NEJMoa2400634. 38838311

[11] de Gooyer PGM, Verschoor YL, van den Dungen LDW, Balduzzi S, Marsman HA, Geukes Foppen MH, et al. Neoadjuvant nivolumab and relatlimab in locally advanced MMR-deficient colon cancer: a phase 2 trial. *Nat Med*. 2024;30(11):3284–90. 10.1038/s41591-024-03250-w. PMC11564102 39278994

[12] Gogenur I, Justesen TF, Tarpgaard LS, Bulut M, Hansen TF, Jensen LH, et al. Neoadjuvant single-cycle pembrolizumab for stage I-III MMR-deficient colon cancer the RESET-C trial. *J Clin Oncol*. 2026;44(18):1687–96. 10.1200/JCO-25-02274. 42085635

[13] de la Fouchardiere C, Zaanan A, de Montfort A, Cohen R, Le Sourd S, Tougeron D, et al. Efficacy of perioperative pembrolizumab in mismatch repair deficient/microsatellite unstable localized colorectal cancers: results of the phase II trial IMHOTEP. *J Clin Oncol*. 2026;44(16):1520–8. 10.1200/JCO-25-02169. 41980235

[14] Wang F, Chen G, Qiu M, Ma J, Mo X, Liu H, et al. Neoadjuvant treatment of IBI310 plus sintilimab in locally advanced MSI-H/dMMR colon cancer: a randomized phase 1b study. *Cancer Cell*. 2025;43(10):1958–67. 10.1016/j.ccell.2025.09.004. 41043438

[15] Loria A, Ammann AM, Olowokure OO, Paquette IM, Justiniano CF. Systematic review of neoadjuvant immunotherapy for mismatch repair deficient locally advanced colon cancer: an emerging strategy. *Dis Colon Rectum*. 2024;67(6):762–71. 10.1097/DCR.0000000000003263. 38479009

[16] Williams CJM, Peddle AM, Kasi PM, Seligmann JF, Roxburgh CS, Middleton GW, et al. Neoadjuvant immunotherapy for dMMR and pMMR colorectal cancers: therapeutic strategies and putative biomarkers of response. *Nat Rev Clin Oncol*. 2024;21(12):839–51. 10.1038/s41571-024-00943-6. 39317818

[17] Mui M, Kong JCH, Michael M, Ramsay R, Clemons NJ, Heriot AG. Role of neoadjuvant immune checkpoint inhibitors in locally advanced rectal cancer: a systematic review of currently available studies. *Dis Colon Rectum*. 2025;68(11):1265–74. 10.1097/DCR.0000000000003927. 40788642

[18] Morton D, Seymour M, Magill L, Handley K, Glasbey J, Glimelius B, et al. Preoperative chemotherapy for operable colon cancer: mature results of an international randomized controlled trial. *J Clin Oncol*. 2023;41(8):1541–52. 10.1200/JCO.22.00046. PMC10022855 36657089

[19] Cottrell TR, Thompson ED, Forde PM, Stein JE, Duffield AS, Anagnostou V, et al. Pathologic features of response to neoadjuvant anti-PD-1 in resected non-small-cell lung carcinoma: a proposal for quantitative immune-related pathologic response criteria (irPRC). *Ann Oncol*. 2018;29(8):1853–60. 10.1093/annonc/mdy218. PMC6096736 29982279

[20] Rousseau B, White JR, Cercek A, Diaz LA Jr. The duration of immunotherapy for mismatch repair-deficient cancers. *N Engl J Med*. 2025;392(8):824–6. 10.1056/NEJMc2409154. PMC12208649 39970404

[21] Sinicrope FA, Ou FS, Arnold D, Peters WR, Behrens RJ, Lieu CH, et al. Atezolizumab plus FOLFOX for stage III mismatch repair-deficient colon cancer. *N Engl J Med*. 2026;394(12):1155–66. 10.1056/NEJMoa2507874. PMC13020640 41880612

[22] Starling N, Elez E, Strickler JH, Benson A, Oki E, Mendez G, et al. A phase III study of perioperative dostarlimab in patients with dMMR/MSI-H resectable colon cancer: AZUR-2 study design. *Future Oncol*. 2026;22(2):137–45. 10.1080/14796694.2025.2606910. PMC12802981 41492912

