# Supplementary material for: Neoadjuvant Immunotherapy in Localized dMMR/MSI-H Colon Cancer: A Systematic Review of Pathological Response and Surgical Outcomes
Source: Ann Surg Oncol. 2026 Aug 17;33(12):10641–50. doi: 10.1245/s10434-026-20430-9 (PMC13675335; doi:10.1245/s10434-026-20430-9)
Supplement: Supplementary file 1 [file 10434_2026_20430_MOESM1_ESM.docx]

| **Database/source** | **Search strategy** |
| --- | --- |
| **Ovid MEDLINE** | ((colon cancer OR colonic cancer OR colorectal cancer OR colon neoplasm* OR colonic neoplasm* OR colorectal neoplasm* OR colon adenocarcinoma OR colonic adenocarcinoma OR colorectal adenocarcinoma) AND (dMMR OR mismatch repair deficient OR mismatch repair deficiency OR MMR deficient OR MMR deficiency OR MSI-H OR microsatellite instability-high OR microsatellite instability high OR microsatellite instability) AND (neoadjuvant OR preoperative OR pre-operative OR perioperative OR peri-operative OR before surgery OR prior to surgery) AND (immunotherapy OR immune checkpoint inhibitor* OR checkpoint inhibitor* OR immune checkpoint blockade OR PD-1 OR PD1 OR programmed cell death-1 OR PD-L1 OR PDL1 OR programmed death ligand-1 OR CTLA-4 OR CTLA4 OR nivolumab OR ipilimumab OR pembrolizumab OR relatlimab OR toripalimab OR sintilimab OR dostarlimab OR camrelizumab OR botensilimab OR balstilimab)) Results: 1217 |
| **Embase** | ((colon cancer OR colonic cancer OR colorectal cancer OR colon neoplasm* OR colonic neoplasm* OR colorectal neoplasm* OR colon adenocarcinoma OR colonic adenocarcinoma OR colorectal adenocarcinoma) AND (dMMR OR mismatch repair deficient OR mismatch repair deficiency OR MMR deficient OR MMR deficiency OR MSI-H OR microsatellite instability-high OR microsatellite instability high OR microsatellite instability) AND (neoadjuvant OR preoperative OR pre-operative OR perioperative OR peri-operative OR before surgery OR prior to surgery) AND (immunotherapy OR immune checkpoint inhibitor* OR checkpoint inhibitor* OR immune checkpoint blockade OR PD-1 OR PD1 OR programmed cell death-1 OR PD-L1 OR PDL1 OR programmed death ligand-1 OR CTLA-4 OR CTLA4 OR nivolumab OR ipilimumab OR pembrolizumab OR relatlimab OR toripalimab OR sintilimab OR dostarlimab OR camrelizumab OR botensilimab OR balstilimab)) Results: 660 |
| **Web of Science** | TS=((“colon cancer” OR “colonic cancer” OR “colorectal cancer” OR “colon neoplasm*” OR “colonic neoplasm*” OR “colorectal neoplasm*” OR “colon adenocarcinoma” OR “colonic adenocarcinoma” OR “colorectal adenocarcinoma”) AND (“dMMR” OR “mismatch repair deficient” OR “mismatch repair deficiency” OR “MMR deficient” OR “MMR deficiency” OR “MSI-H” OR “microsatellite instability-high” OR “microsatellite instability high” OR “microsatellite instability”) AND (“neoadjuvant” OR “preoperative” OR “pre-operative” OR “perioperative” OR “peri-operative” OR “before surgery” OR “prior to surgery”) AND (“immunotherapy” OR “immune checkpoint inhibitor*” OR “checkpoint inhibitor*” OR “immune checkpoint blockade” OR “PD-1” OR “PD1” OR “programmed cell death-1” OR “PD-L1” OR “PDL1” OR “programmed death ligand-1” OR “CTLA-4” OR “CTLA4” OR “nivolumab” OR “ipilimumab” OR “pembrolizumab” OR “relatlimab” OR “toripalimab” OR “sintilimab” OR “dostarlimab” OR “camrelizumab” OR “botensilimab” OR “balstilimab”)) Results: 478 |
| **Cochrane Library / CENTRAL** | ((colon cancer OR colonic cancer OR colorectal cancer OR colon neoplasm* OR colonic neoplasm* OR colorectal neoplasm* OR colon adenocarcinoma OR colonic adenocarcinoma OR colorectal adenocarcinoma) AND (dMMR OR mismatch repair deficient OR mismatch repair deficiency OR MMR deficient OR MMR deficiency OR MSI-H OR microsatellite instability-high OR microsatellite instability high OR microsatellite instability) AND (neoadjuvant OR preoperative OR pre-operative OR perioperative OR peri-operative OR before surgery OR prior to surgery) AND (immunotherapy OR immune checkpoint inhibitor* OR checkpoint inhibitor* OR immune checkpoint blockade OR PD-1 OR PD1 OR programmed cell death-1 OR PD-L1 OR PDL1 OR programmed death ligand-1 OR CTLA-4 OR CTLA4 OR nivolumab OR ipilimumab OR pembrolizumab OR relatlimab OR toripalimab OR sintilimab OR dostarlimab OR camrelizumab OR botensilimab OR balstilimab)) Results: 81 |
| **ClinicalTrials.gov** | (colon cancer OR colorectal cancer) AND (dMMR OR MSI-H OR microsatellite instability OR mismatch repair deficient) AND (neoadjuvant OR preoperative OR perioperative) AND (immunotherapy OR immune checkpoint inhibitor OR PD-1 OR PD-L1 OR CTLA-4 OR nivolumab OR ipilimumab OR pembrolizumab OR relatlimab OR toripalimab OR sintilimab OR dostarlimab OR camrelizumab) Results: 69 |

Supplementary Table 1: Search Strategy

| **Study** | **Clearly stated aim** | **Consecutive patients** | **Prospective data collection** | **Appropriate endpoints** | **Unbiased endpoint assessment** | **Follow-up appropriate** | **Loss to follow-up <5%** | **Prospective sample size** | **Control group** | **Contemporary groups** | **Baseline equivalence** | **Adequate statistical analysis** | **Total score** | **Quality rating** |
| --- | --- | --- | --- | --- | --- | --- | --- | --- | --- | --- | --- | --- | --- | --- |
| **NICHE** | 2 | 2 | 2 | 2 | 2 | 1 | 2 | 1 | NA | NA | NA | NA | **14/16** | High |
| **NICHE-2** | 2 | 2 | 2 | 2 | 2 | 2 | 2 | 2 | NA | NA | NA | NA | **16/16** | High |
| **NICHE-3** | 2 | 2 | 2 | 2 | 2 | 1 | 2 | 2 | NA | NA | NA | NA | **15/16** | High |
| **RESET-C** | 2 | 2 | 2 | 2 | 1 | 1 | 2 | 2 | NA | NA | NA | NA | **14/16** | High |
| **IMHOTEP** | 2 | 2 | 2 | 2 | 1 | 2 | 2 | 2 | NA | NA | NA | NA | **15/16** | High |
| **Wang et al.** | 2 | 2 | 2 | 2 | 2 | 2 | 2 | 2 | 1 | 2 | 2 | 2 | **23/24** | High |

Supplementary Table 2. Methodological quality assessment using the Methodological Index for Non-Randomized Studies.

Non-comparative studies were scored out of 16 and comparative or parallel-arm studies out of 24. Wang et al. was scored using the comparative MINORS framework because it used random allocation to two treatment arms. |
Lower scores for follow-up reflect immature follow-up relative to long-term oncological endpoints. MINORS scoring: 0, not reported; 1, reported but inadequate; 2, reported and adequate.

Quality categories: for non-comparative studies, 0–4 very low quality, 5–8 low quality, 9–12 moderate quality, and 13–16 high quality. For comparative studies, 0–6 very low quality, 7–12 low quality, 13–18 moderate quality, and 19–24 high quality.

Abbreviations: MINORS, Methodological Index for Non-Randomized Studies; NA, not applicable.

| **Study** | **Any treatment-related / immune-related AE** | **Grade ≥3 treatment-related / immune-related AE** | **Treatment discontinuation** | **Surgical delay due to toxicity** | **Postoperative morbidity** | **Anastomotic leak** | **Treatment-related death** |
| --- | --- | --- | --- | --- | --- | --- | --- |
| **NICHE** | NR overall in table format | Grade 3–4 treatment-related toxicity 5/40 overall cohort | NR | None reported | Surgery-related grade 3 AEs 8/40 overall cohort | 4/40 overall cohort | 0 |
| **NICHE-2** | irAEs reported; detailed categories in supplement | Grade 3–4 irAEs 5/115 | 0 due to AEs | >2-week surgical delay in 2/115 | Surgery-related AEs 22/115; grade 3 surgery-related AEs 12/115 | 4/115 | 0 |
| **NICHE-3** | Any-grade irAE 47/59 | Grade 3–4 irAE 6/59 | NR | Treatment-related surgical delay in 3/59 | Grade 3–4 postoperative AEs 4/59 | 1/59 | 0 |
| **RESET-C** | Any AE 70/85 | Grade 3 AEs 9/85; three treatment-related grade 3 events | NR | NR | Surgical complications 31/84; Clavien-Dindo ≥3b 8/84 | NR | 0 treatment-related; two deaths within 30 days of surgery |
| **IMHOTEP** | TRAE any grade 67/89 | Grade ≥3 TRAE 14/89 | Pembrolizumab discontinued in some patients due to AEs | NR | Postoperative Clavien-Dindo complications 13/78 operated patients | NR | 1 grade 5 pembrolizumab-related myasthenia |
| **Wang et al.** | TRAEs: 37/52 combination; 24/49 monotherapy. irAEs: 25/52 combination; 19/49 monotherapy | Grade ≥3 TRAEs: 2/52 combination; 5/49 monotherapy. Grade ≥3 irAEs: 2/52 combination; 4/49 monotherapy | 0 combination; 1/49 monotherapy | 2/52 combination; 0 monotherapy due to TRAE | NR in main outcomes table | NR | 1 grade 5 immune-mediated myocarditis in monotherapy arm |

Supplementary Table 3. Detailed treatment-related and perioperative safety outcomes.

Adverse-event definitions and reporting windows varied across studies. Neoadjuvant-only and perioperative treatment strategies are reported as described in the original studies; perioperative studies may capture adverse events occurring beyond the preoperative treatment window. Abbreviations: AE, adverse event; irAE, immune-related adverse event; NR, not reported; TRAE, treatment-related adverse event.
